# Competitive fitness and stability of ammonium-excreting *Azotobacter vinelandii* strains in the soil

**DOI:** 10.1007/s00253-024-13231-1

**Published:** 2024-06-18

**Authors:** Rafael Ambrosio, Gonzalo Burgos Herrera, Mauro Do Nascimento, Luciana Anabella Pagnussat, Leonardo Curatti

**Affiliations:** 1 Instituto de Investigaciones en Biodiversidad y Biotecnología (INBIOTEC), Consejo Nacional de Investigaciones Científicas y Técnicas, Vieytes 3103, 7600 Mar del PlataBuenos Aires, Argentina; 2 Fundación para Investigaciones Biológicas Aplicadas, Mar del Plata, Buenos Aires Argentina; 3 Instituto de Agrobiotecnología del Litoral, Consejo Nacional de Investigaciones Científicas y Técnicas, Universidad Nacional del Litoral, Santa Fe, Santa Fe Argentina; 4 Facultad de Ciencias Agrarias, Universidad Nacional de Mar del Plata, Balcarce, Buenos Aires Argentina

**Keywords:** N-fertilizer, Sustainable agriculture, Nitrogen fixation, Inoculants, Bacterial fitness

## Abstract

**Abstract:**

Non-symbiotic N_2_-fixation would greatly increase the versatility of N-biofertilizers for sustainable agriculture. Genetic modification of diazotrophic bacteria has successfully enhanced NH_4_^+^ release. In this study, we compared the competitive fitness of *A. vinelandii* mutant strains, which allowed us to analyze the burden of NH_4_^+^ release under a broad dynamic range. Long-term competition assays under regular culture conditions confirmed a large burden for NH_4_^+^ release, exclusion by the *wt* strain, phenotypic instability, and loss of the ability to release NH_4_^+^. In contrast, co-inoculation in mild autoclaved soil showed a much longer co-existence with the *wt* strain and a stable NH_4_^+^ release phenotype. All genetically modified strains increased the N content and changed its chemical speciation in the soil. This study contributes one step forward towards bridging a knowledge gap between molecular biology laboratory research and the incorporation of N from the air into the soil in a molecular species suitable for plant nutrition, a crucial requirement for developing improved bacterial inoculants for economic and environmentally sustainable agriculture.

**Key points:**

*• Genetic engineering for NH*_*4*_^*+*^ *excretion imposes a fitness burden on the culture medium*

*• Large phenotypic instability for NH*_*4*_^*+*^ *-excreting bacteria in culture medium*

*• Lower fitness burden and phenotypic instability for NH*_*4*_^*+*^ *-excreting bacteria in soil*

**Supplementary Information:**

The online version contains supplementary material available at 10.1007/s00253-024-13231-1.

## Introduction

Current and future food security demands higher crop yields, which are largely dependent on N-fertilizers (Bodirsky et al. 2014). The Green Revolution of the 1960s was mainly enabled by the development of semi-dwarf cereal varieties, which resulted in substantially higher grain yields. However, most of these varieties have a lower N use efficiency (yield per unit of nitrogen N-fertilizer input) than their parental wild-type varieties, and thus, they require a higher N-fertilizer input to realize the potential for high grain yields (Liu et al. 2022). As a consequence, several detrimental aspects are associated with this fact: (1) poorest regions of the world have limited access to N-fertilizers at global prices, which jeopardizes food security (He et al. 2021); (2) current industrial production of synthetic N-fertilizer by the Haber–Bosch process represents the most energy-intensive commodity chemical, responsible for 1–2% of global energy consumption and 1.44% of CO_2_ emissions (Kyriakou et al. 2020); and (3) due to incorrect agricultural management, including improper dosing and timing of fertilizer application, a significant portion of the applied nitrogen fertilizer persists in the environment (Ladha et al. 2016), producing a variety of health adverse conditions and environmental odds (Kanter et al. 2020).

A 50-year-period analysis (1961 to 2010) indicated that 48% of N harvested as cereal grain was supplied as synthetic N-fertilizer, 24% of the harvested N could be traced to non-symbiotic bacterial N_2_-fixation, and the rest corresponded to manure (16%), atmospheric deposition (6%), and net soil depletion (4%) (Ladha et al. 2016).

Promoting bacterial nitrogen fixation (BNF) in agriculture has long been considered one of the most promising alternatives to synthetic nitrogen fertilizers. BNF contributes to sustainable agriculture by providing both affordability and environmental benefits. N_2_-fixing bacteria can turn atmospheric N_2_ into ammonia by means of the complex enzyme nitrogenase (Einsle and Rees 2020). Symbiotic BNF between some leguminous plants and rhizobia represents a very successful application of BNF in sustainable agriculture. These bacterial-plant associations rely on very specific and sophisticated partner-recognition signaling at the molecular level, enabling a productive interaction between the symbionts, in which the bacterial partner fixes N_2_ for itself and also for the plant host (Lindström and Mousavi 2020). Conversely, non-symbiotic BNF is performed by diazotrophic bacteria with a wide spectrum of associativeness with plants, from closely associated endophytes to free-living bacteria (Roper and Gupta 2016). On one hand, the lower to null plant partner specificity would increase largely the versatility of these N-biofertilizers. However, the extent to which the current contribution of these bacteria to plant nitrogen nutrition can be realistically enhanced further remains a topic of debate (Kennedy et al. 2004; Lindström and Mousavi 2020). This is in part because free-living and endophytic bacteria regulate intracellular N-homeostasis very tightly at different levels of N_2_-fixation and N-assimilation and do not release enough N to sustain industrial crop productivities (Smercina et al. 2019; Bueno Batista and Dixon 2019). Enhancement of NH_4_^+^ release was successfully achieved by genetic modification of the model bacterium *Azotobacter vinelandii*, and other diazotrophic bacteria (Bueno Batista and Dixon 2019; Barney 2020). Three strategies were mainly used in *A. vinelandii* to fulfill that goal: (i) disruption of the NifA/NifL-dependent NH_4_^+^ control of the *nif* gene expression (Bali et al. 1992; Ortiz-Marquez et al. 2012; Barney et al. 2015), (ii) partial inhibition of glutamine synthetase (GS) for deficient NH_4_^+^ assimilation (Ortiz-Marquez et al. 2014; Ambrosio et al. 2017), and (iii) disruption of the ammonium/methyl ammonium transporter AmtB (Barney et al. 2015).

Inoculation of plants with some of these genetically modified *A. vinelandii* strains substituted for at least some synthetic N-fertilizer for plant growth (Ambrosio et al. 2017; Bageshwar et al. 2017; Mus et al. 2022). Similar results were obtained using other bacterial species similarly modified (Fox et al. 2016; Santos et al. 2017; Schnabel and Sattely 2021a). Under laboratory growth conditions, NH_4_^+^ excretion is considerably more pronounced in mutant strains and/or under conditions when cell growth and biomass production slow down (Bali et al. 1992; Ortiz-Marquez et al. 2012; Ortiz-Marquez et al. 2014; Ambrosio et al. 2017; Ambrosio and Curatti 2021). Accordingly, both in *A. vinelandii* (Ortiz-Marquez et al. 2012; Barney et al. 2015; Ambrosio and Curatti 2021) and other diazotrophic bacteria (Schnabel and Sattely 2021a; Schnabel and Sattely 2021b), the NH_4_^+^-excreting phenotype proved to be genetically unstable and frequently lost after a few bacterial generations during cultivation under laboratory conditions (Ortiz-Marquez et al. 2012; Barney et al. 2015; Ambrosio and Curatti 2021; Schnabel and Sattely 2021a; Schnabel and Sattely 2021b).

Natural ecosystems, such as the soil, present variable resistance to invasion by alien species, including inoculant microorganisms, by a mechanism that in part depends on the native species diversity (van Elsas, et al 2012). Thus, poor growth and genetic instability, in the context of soil microbial ecology, raise some questions regarding the realistic potential of bacterial genetic engineering of NH_4_^+^ release for sustainable crop N-fertilization.

In this study, we performed a competitive fitness analysis of *A. vinelandii* strains genetically modified at different levels to excrete NH₄⁺. We compared these modified strains to the non-gummy laboratory strain DJ, as the reference strain in this study. The analysis was conducted under reference laboratory culture conditions and using soil that underwent a gentle autoclaving process. Results show a complex interaction under a competitive scenario. While regular culture conditions led to a large burden for NH_4_^+^-release and phenotypic stability, mutant strains analyzed proved to be more stable and efficient at increasing the mineral-N content of the experimental soil after 2 months of inoculation.

## Materials and methods

### Microorganisms and submerged culture in regular culture medium

**Fig. 1:**
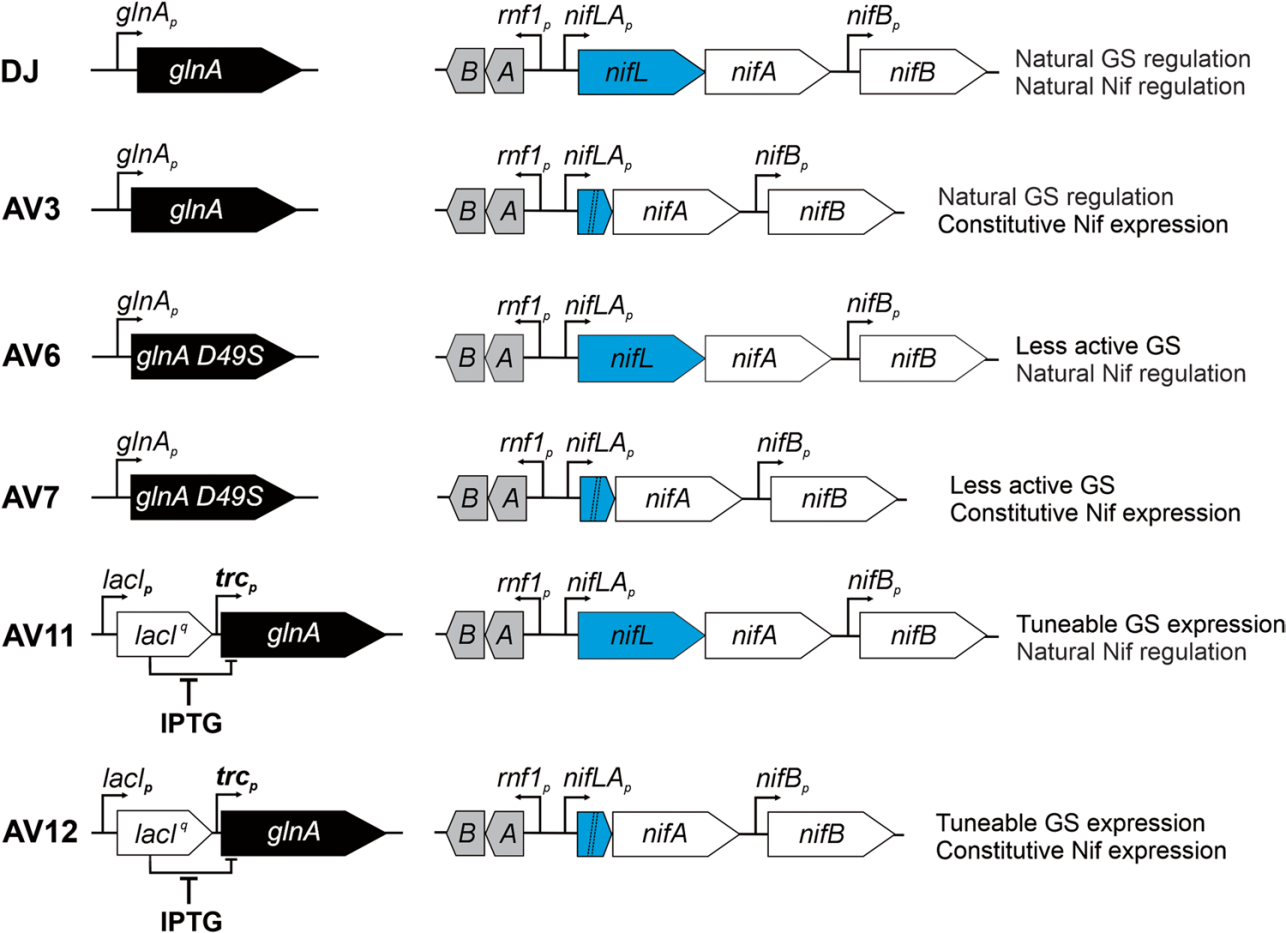
Genetic background of the strains used in this study. DJ represents the *wt* strain

The reference strain in this study was *Azotobacter vinelandii* (*wt*), strain DJ (ATCC BAA-1303). Isolation of mutant strains AV3 (*ΔnifL*) (Ortiz-Marquez et al. 2012), AV6 (*glnA D49S*) and AV7 (*ΔnifL*; *glnA D49S)* (Ortiz-Marquez et al. 2014), and AV11 (*trc*_*P*_*-glnA*) and AV12 (*trc*_*P*_*-glnA*; Δ*nifL*) (Ambrosio et al. 2017) was previously described. Figure 1 shows the genetic background and main characteristics of these strains. AV11 and AV12 inocula were prepared onto plates containing 0.3 mM isopropyl β-d-1-thiogalactopyranoside (IPTG) for the induction of *glnA* for 3 days at 29 ± 1 °C and then in liquid medium supplemented with the indicated concentration of IPTG for 2 days. The *wt* strain was also labeled with a rifampicin-resistant allele from pDB303 (D. Dean), to facilitate the identification of this strain (DJ-Rif^R^) in competitive fitness analysis. DJ-Rif^R^ grew as fast as DJ under regular culture conditions. Transformation of *A. vinelandii* was conducted essentially as indicated before (Page and Von Tigerstrom 1979).

Cultures under reference laboratory conditions were Burk’s medium (Newton et al. 1953) using atmospheric N_2_ as the sole N-source and shaking at 180 rpm at 29 ± 1 °C.

### *Azotobacter vinelandii* population dynamics in poor soil

For cultivation in soil, two different approaches were followed. The “homogenized soaked soil approach” was intended to allow some mixing and multiple sampling from the same pot/tube for simplified time-course analysis. The soil substrate was prepared by mixing perlite and sand in a 1:1 (v/v) ratio. Then, 12.5% (v/v) of fertile garden soil was added. Fifty-mL centrifuge tubes were loaded with 40 g of the soil substrate and autoclaved at 121 °C for 20 min. When indicated, 1.5 mg of C per gram of soil substrate was added every other 8 days using a sterile solution of 0.5-M glucose. For the preparation of these inocula, all bacterial strains were cultivated in Burk’s medium supplemented with 29-mM ammonium acetate for 16 h. Additionally, the *trcP-glnA* strains were loaded with the IPTG concentration indicated in each experiment for two sub-cultivation runs of 24 h each. All cultures were washed with NH_4_^+^-free medium prior to inoculation. Tubes were incubated at 22 ± 2 °C and mixed by gentle shaking every other 4 days. Corrections were made based on the difference in weight for the addition of glucose. Sampling was performed by withdrawing 0.3 to 0.7 g soil substrate right after a mixing occasion. These samples were suspended in an appropriate volume of sterile phosphate solution from the Burk’s medium for bacterial CFU counting and NH_4_^+^ determination (see below).

The “undisturbed soil approach” was envisioned for a more realistic analysis of the dynamics of bacterial populations and N accumulation and chemical speciation in the soil. The soil substrate consisted of a mixture of washed river sand, perlite, and soil collected from the upper 10 cm of a typical Argiudoll (5.4% organic matter; 11.5 ppm P; 8.85 ppm NO_3_^−^-N; pH 5.7) in a 45:45:10 ratio and was autoclaved at 121 °C for 20 min. When required, certain pots received weekly supplementation with a 1 ml solution containing 0.02 moles of glucose per gram of soil substrate at a depth of 1.5 cm. For the assays, 50-mL or 200-mL pots were filled up to the top with the soil substrate for the determination of bacterial CFU or N-content, respectively. Pots were placed in a growth chamber at 24 °C with a light/dark cycle of 16:8 h and watered once or twice a week with distilled water (about 100 mL per week) maintaining field capacity since a week before inoculation and throughout the entire experiment. Pots were inoculated once with 2 **·** 10^7^ bacterial CFU **·** g^−1^ soil substrate on day 1, and samples were taken on day 28 or 77, for CFU or N determinations, respectively. For this, the specific relationship between OD_600_ and CFU for each strain was previously determined. Bacterial inocula were prepared as described before. A 1-mL suspension of the indicated strain, or combination of strains, was inoculated in the center of each pot at a depth of 1.5 cm. For CFU determinations, the whole soil content from the 50-mL pots was thoroughly homogenized with 50 mL of sterile saline solution, and the bacterial CFU **·** g^−1^ soil count was determined.

For N chemical species determinations, sample preparation involved drying the entire soil content from the 200-mL pots at 60 °C.

### Analytical methods

Bacterial CFU was determined using the microdrop technique (Herigstad et al. 2001). The antibiotics used were rifampicin (Rif), ampicillin (Ap), and kanamycin (Kn). Serial dilutions were seeded onto Burk’s medium containing 5 µg **·** mL^−1^ Rif (for DJ-Rif^R^), 50 µg **·** mL^−1^ Ap (for AV6) or 50 µg **·** mL^−1^ Ap, and 5 µg **·** mL^−1^ Kn and 0.3 mM IPTG (for AV11 and AV12), and plates were incubated at 28 °C. For samples containing a mixture of *A. vinelandii* DJ and a mutant strain, each strain was ascertained separately according to its antibiotic resistance. *Azotobacter* colonies were accurately ascertained, among other developing soil bacteria and fungi when plates were allowed to develop for 3–4 days, and observed under an illuminated binocular magnifying glass.

For the determination of the total N, NH_4_^+^, and NO_3_^−^, analyses were performed by a specialized laboratory (Soil Analysis Laboratory INTA. EEA Balcarce, Argentina; https://www.argentina.gob.ar/inta) after steam-distillation according to Bremner and Keeney (1966).

In-house NH_4_^+^ determinations were performed using the indophenol method using an NH_4_Cl standard, essentially as reported before (Ortiz-Marquez et al. 2012).

Variance analysis was performed using the software GraphPad Prism version 8.0 (http://www.graphpad.com/). The variables were compared by the minimum differences test or Tukey as appropriate.

## Results

### Competitive fitness of *A. vinelandii* NH_4_^+^-excreting strains under reference laboratory conditions

To compare the growth and NH_4_^+^ release of *A. vinelandii* NH_4_^+^-excreting strains due to mutations in *nifL* and/or *glnA* (Fig. 1) under reference laboratory conditions, mutant cells were withdrawn daily from cultures at logarithmic phase and re-inoculated into fresh medium for NH_4_^+^ removal and nutrient replenishment for up to 7 consecutive days (sub-cultures). This enabled us to extend the analysis for many more generations than normally achieved from single-growth curve analysis.

**Fig. 2:**
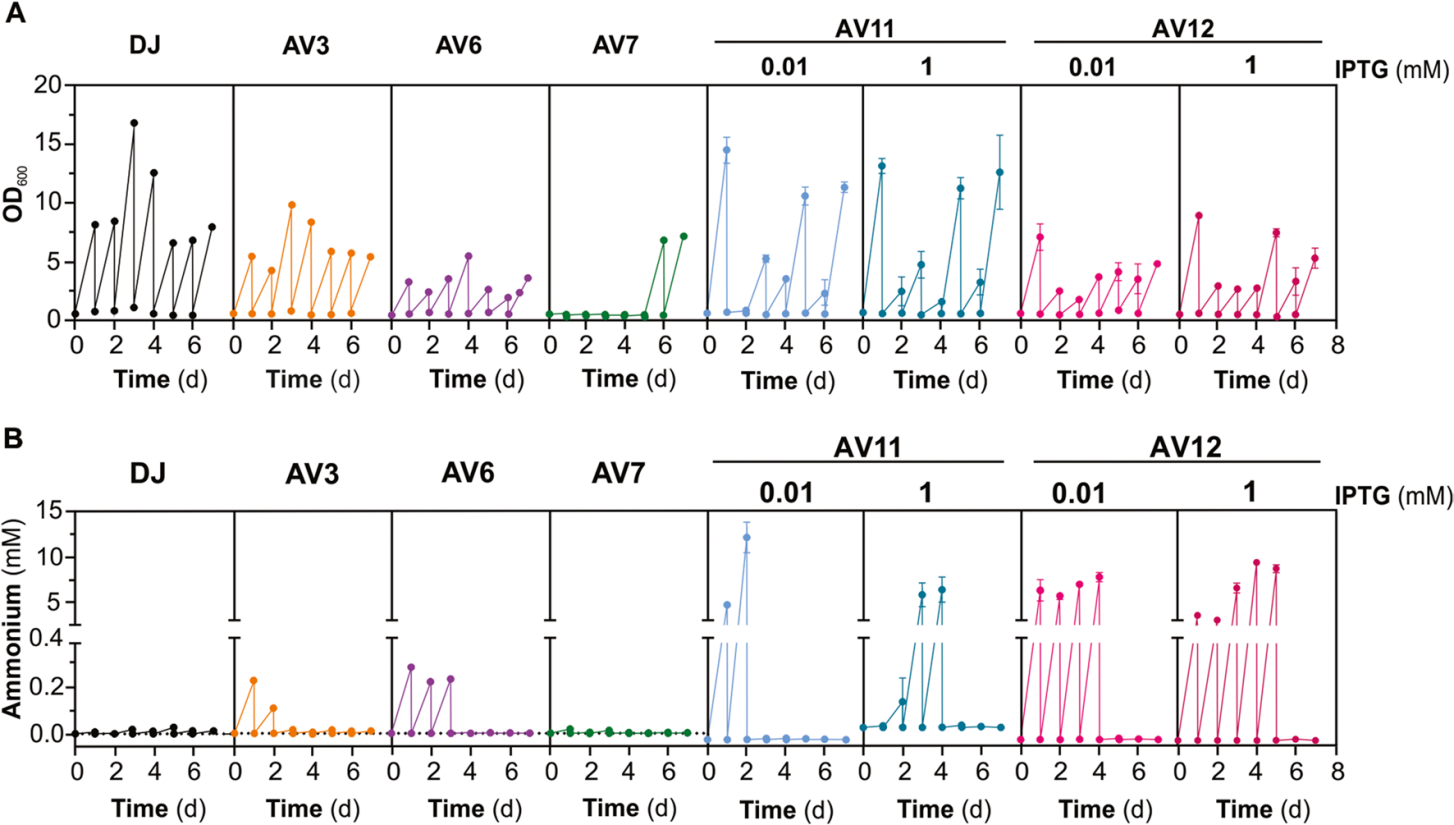
Phenotypic stability of different NH_4_^+^-excreting *A. vinelandii* mutant strains in regular culture medium. Cells of *A. vinelandii* strains were washed and diluted to the initial optical density into fresh medium every other 24 h for seven consecutive sub-culture cycles. **A** Growth (OD_600_) and **B** NH_4_^+^ accumulation at the end of each sub-culture cycle. Each data point represents a single representative experiment for DJ, AV3, AV6, and AV7 and the mean and standard error of two independent experiments for strains AV11 and AV12

Figure 2 shows the typical profiles of growth (Fig. 2A) and NH_4_^+^ release (Fig. 2B) of different mutant strains. While AV3 (*ΔnifL*) and AV6 (*glnA D49S*) grew consistently slower than the parental stain DJ, the double mutant strains AV7 (*ΔnifL*; *glnA D49S*) grew extremely slowly until the 6th sub-culture, when it began to grow almost as fast as the *wt* strain. On the other hand, AV11 (*trc*_*P*_*-glnA*) previously induced to over-accumulate glutamine synthetase (GS) grew even faster than strain DJ during the first sub-cultures until growth declined as a consequence of GS depletion in the non-inducing medium. However, as demonstrated previously (Ambrosio and Curatti 2021), after several generations, it tends to revert to growing at the same rate as glutamine synthetase-replete cells. Specifically, AV12 (*trc*_*P*_*-glnA*; Δ*nifL*) exhibited slower growth than AV11, and the inclination to revert to faster-growing cells was less pronounced.

All the mutant strains excreted NH_4_^+^ at considerably different levels during the first sub-cultures, but all of them stopped releasing NH_4_^+^ beyond the 5th sub-culture. AV11 (*trc*_*P*_*-glnA*) showed the previously observed IPTG-dependent delay in NH_4_^+^ excretion, which is lost by an additional mutation on the *nifL* in strain AV12 (Ambrosio and Curatti 2021). Overall, AV12 (*trc*_*P*_*-glnA*; Δ*nifL*) appeared to be the most stable strain of this study in terms of growth and NH_4_^+^ release over time.

To develop a simplified model of bacterial competition in the environment, we conducted several competition assays by co-cultivating each NH_4_^+^-excreting strain with the *wt* strain (DJ-Rif^R^) in competition assays. The experimental setting was designed to ameliorate competence by continuously replenishing nutrients and removing metabolic waste products.

**Fig. 3:**
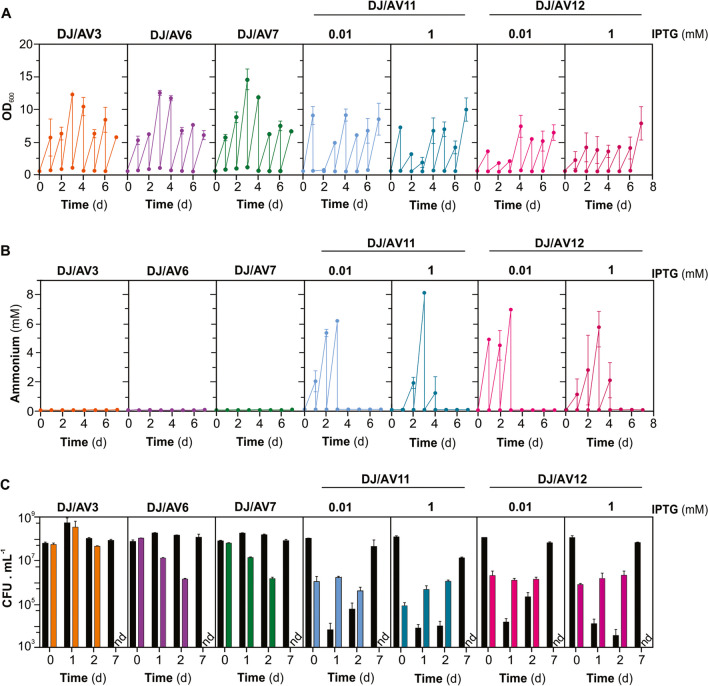
Relative competitive fitness of different NH_4_^+^-excreting *A. vinelandii* mutant strains in a regular culture medium. For the assays shown in this figure, strains DJ-Rif^R^ (*wt*), AV3 (*∆nifL*), AV6 (*glnA D49S*), and AV7 (*∆nifL*; *glnA D49S*) were initially inoculated at about 5 **·** 10^7^ CFU mL and AV11 (*trcP::glnA*) and AV12 (*trcP::glnA*;* ∆nifL* ) at about 1 **·** 10^6^ CFU mL. Cells were washed and diluted to the initial optical density into fresh medium every other 24 h for seven consecutive sub-culture cycles. **A** Growth (OD_600_), **B** NH_4_^+^ accumulation at the end of each sub-culture cycle, and **C** CFU determinations at the beginning and end of cycles one, two, and seven. Each strain was identified according to its unique selection marker. Each data point represents the mean and standard error of two independent experiments

As expected, the presence of the DJ-Rif^R^ strain, mimicking any microbe in the soil, prevented NH_4_^+^ accumulation in the culture medium at any time for co-cultures including AV3 (*ΔnifL*), AV6 (*glnA D49S*), or AV7 (*ΔnifL*; *glnA D49S*). However, co-cultures comprising AV11 (*trcP-glnA*) or AV12 (*trcP-glnA*; *ΔnifL*) still accumulated ammonium, albeit at a lower level than monocultures of the mutant strains (Fig. 3B). This appears to be coincidental with a marked decline on the *wt* population during the first growth cycles of co-cultures (Fig. 3C).

In agreement with the strains’ relative growth rate in monoculture, competition with the DJ-Rif^R^ strain produced a rapid and quite dramatic decline of about 100-fold of the population of AV6 (*glnA D49S*) or AV7 (*ΔnifL*; *glnA D49S*) at the second day. AV3 (*ΔnifL*) displayed a similar fitness to the *wt* strain in the short term, up to the second sub-culture, and declined afterward up to below the assay detection limit by the end of the experiment. Interestingly, strains AV11 (*trcP-ΔglnA*) and AV12 (*trcP-glnA*; *ΔnifL*) exhibited significantly higher competitive fitness compared to the DJ-Rif^R^ strain. As a consequence, the population of the wild-type strain declined by up to 10,000-fold by the second day, despite the fact that in these experiments, its initial ratio was 100-fold lower than that of DJ-Rif^R^. This increased relative fitness appeared to be dependent on the previous accumulation level of glutamine synthase in these mutant stains (Fig. 3C). However, at longer times (7th sub-culture), the *wt* strain largely overgrew the mutant strains. The CFU counts for the mutants remained below the detection limit of the assay (10^3^ CFU **·** mL^−1^). Meanwhile, the *wt* cells recovered their original CFU count at the beginning of the experiment (time, 0 days) (Fig. 3C). In an independent experiment, we replicated the setup shown in Fig. 3. However, this new experiment employed more evenly distributed initial populations of DJ-Rif^R^ and AV11 (*trcP-glnA*) strains. Furthermore, we conducted a detailed analysis of the initial accumulation level of glutamine synthetase in AV11 (*trcP-glnA*). Remarkably, the results from this experiment matched those of the previous observation, thereby confirming its validity (Supplementary Fig. S1).

#### *Azotobacter vinelandii* population dynamics and effects on N content and chemical speciation in the soil

In the field of environmental microbiology, demonstrating the potential applications of microbial processes under controlled conditions is crucial. However, due to the inherent complexity of soil ecosystems, conducting experiments in a fully controlled environment is rarely feasible as an initial approach for soil microbiology research. Therefore, preliminary or partial experiments are necessary to begin unraveling the intricate dynamics of soil microbial ecology (Dietrich et al. 2020). Soil sterilization, whether partial or complete, serves as a fundamental tool for investigating the ecological roles of soil biota. While complete sterilization (e.g., through autoclavation or high-dose gamma irradiation) allows maximum control over the microbial community, it results in simplified communities that do not fully mirror the diversity found in natural soils. Additionally, it alters the physicochemical characteristics of the soil. As an alternative, partial or selective sterilization is often preferred. This approach minimizes changes to both biotic and abiotic soil properties, maintaining a more realistic experimental system while still allowing some control (Hassi et al. 2023; Querejeta 2023; Mocali et al. 2022).

In this study, we initially conducted preliminary experiments by inoculating the parental *A. vinelandii* strain (DJ-Rif^R^) into non-treated soil. However, excessive microbial development—particularly fungi—on the regular *Azotobacter* culture medium hindered unequivocal strain identification beyond 1 month. Despite adding cycloheximide and/or antibiotics to the plates, the situation did not improve significantly (not shown). Consequently, we chose a mild autoclaving treatment of the soil at 120 °C for 20 min. This treatment allowed us to count the parental strain after several weeks. Initially, we speculated that this condition would also enable the counting of mutant strains, some of which exhibited a severe slowdown in their growth rate. Additionally, we employed two complementary experimental approaches: (1) a “homogenized soaked soil approach” which consisted semi-continuously mixing soil substrate, air, and bacteria. It allowed for multiple samplings from the same pot, simplifying time-course analysis. And (2) an “undisturbed soil approach” which consisted of a single-point inoculation without any mixing. This approach provided a more realistic scenario for assessing bacterial stability and competence, and N accumulation and its chemical speciation in the soil.

### Homogenized soaked soil approach

**Fig. 4:**
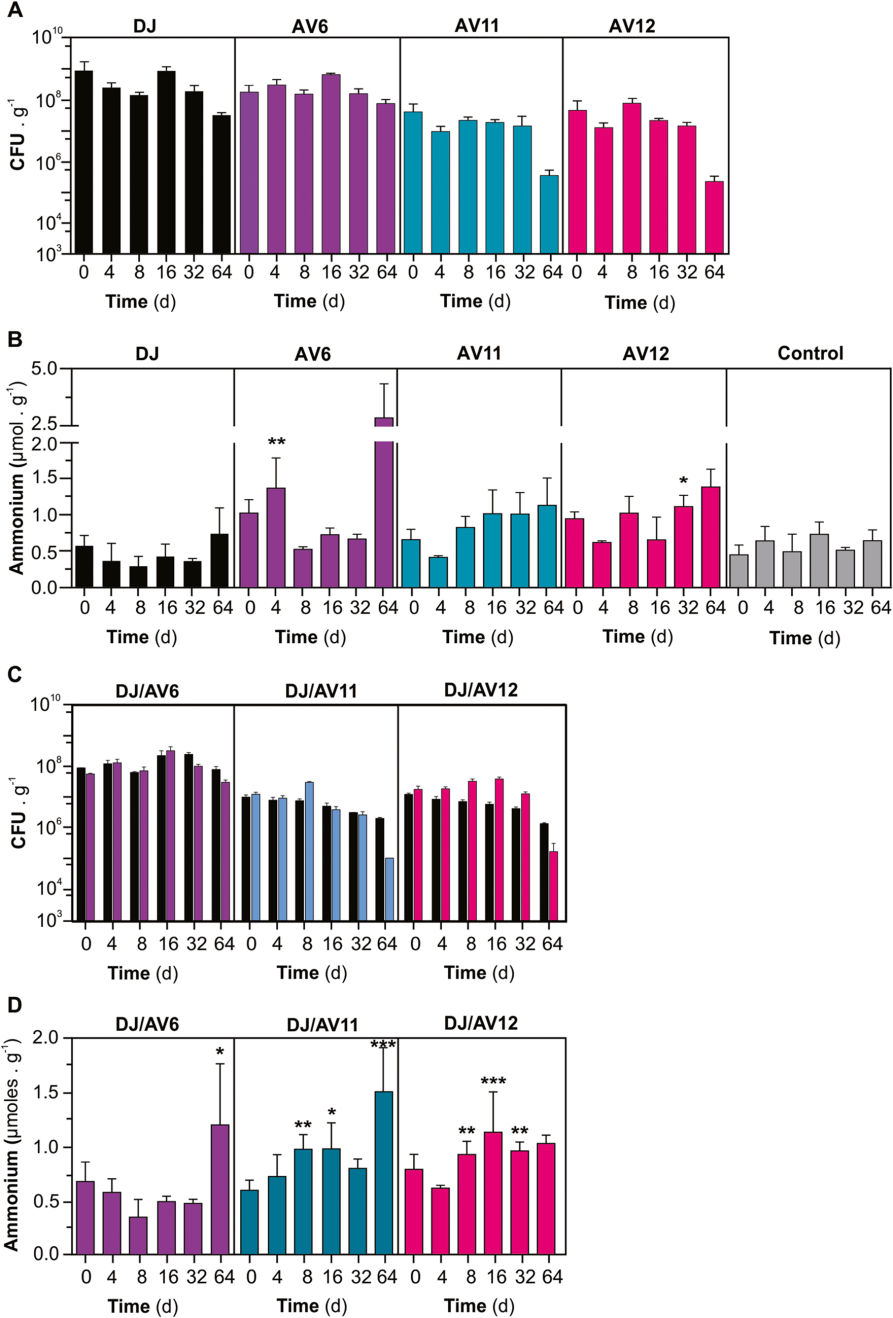
Survival, relative competitive fitness, and NH_4_^+^ release of different *A. vinelandii* mutant strains in semi-continuously homogenized soil. Initial CFU were about 1 **·** 10^7^ to 1 **·** 10^8^ **·** mL^−1^. **A** Time course of CFU recovery from the soil. **B** Time course of NH_4_^+^ accumulation in the soil after inoculation with individual strains; hyphens (-) indicate non-inoculated soils. **C** Time course of the relative competitive fitness of *A. vinelandii* DJ-Rif^R^ and mutant strains in the soil. **D** Time course of NH_4_^+^ accumulation in soil after inoculation with combinations of *wt* and mutant strains. Each data point represents the mean and standard deviation of three independent experiments. Results were statistically analyzed using ANOVA and Tukey as multiple comparison posttests between each treatment and non-inoculated at each sampling time. Asterisks (*) in **B** and **D** indicate significant differences (*p ≤ 0.05; *p ≤ 0.01; **p ≤ 0.001; and **p ≤ 0.0001)

CFUs of each strain were analyzed at different time points for up to 64 days after inoculation. Figure 4A shows that the population of DJ-Rif^R^ and AV6 (*glnA D49S*) only decreased moderately (less than tenfold) by the end of the experiment. Conversely, AV11 (*trcP-glnA*) and AV12 (*trcP-glnA*; *ΔnifL*) declined more sharply (about 1000-fold) at the same time. This was expected because the soil was not supplemented with IPTG.

Inoculation with DJ-Rif^R^ resulted in a slight (but non-statically significant) decrease in the NH_4_^+^ content of the soil. On the other hand, the AV12 (*trcP-glnA*; *ΔnifL*) mutant strain increased the NH_4_^+^ soil levels by 214.4% (*p = 0.046*) after 32 days in comparison to the control, and after 64 days, the AV6 (*glnA D49S*) strain increased the NH_4_^+^ soil level by 339.4% (*p < 0.0001*) (Fig. 4B).

When DJ-Rif^R^ was co-inoculated, there was no significant change in the recovery of the mutants’ CFUs compared to monoculture inoculations (Fig. 4C). However, it did not completely prevent the accumulation of NH₄⁺ in the soil, although the level was lower than that observed in monocultures (Fig. 4D).

**Fig. 5:**
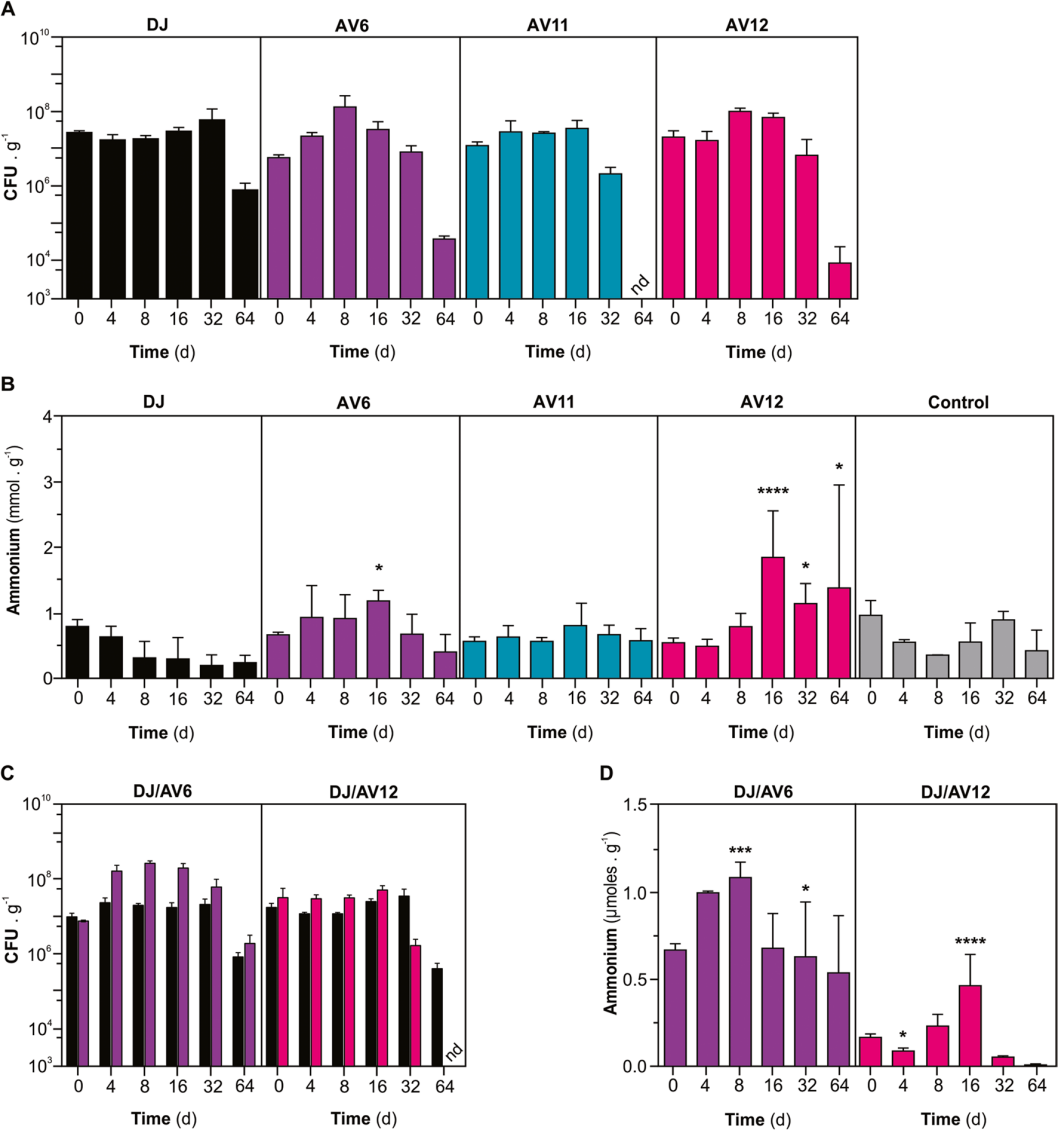
Survival, relative competitive fitness and NH_4_^+^ release of different *A. vinelandii* mutant strains in semi-continuously homogenized soil supplemented with glucose. Initial CFU was about 1 **·** 10^7^ **·** mL^−1^. **A** Time course of CFU recovery from the soil. **B** Time course of NH_4_^+^ accumulation in the soil after inoculation with individual strains; hyphens (-) indicate non-inoculated soils. **C** Time course of the relative competitive fitness of *A. vinelandii* DJ-Rif^R^ and mutant strain in the soil. **D** Time course of NH_4_^+^ accumulation in soil after inoculation with combinations of *wt* and mutant strains. Each data point represents the mean and standard deviation of three independent experiments

To experimentally simulate rhizospheric conditions, the soil was supplemented at regular intervals with glucose at 1.5 mg C **·** g^−1^ soil substrate every other 8 days. Glucose addition enabled a moderate increase in the population of most strains, especially DJ-Rif^R^ and AV6 (*glnA D49S*). However, all strains declined sharply (more than 1000-fold) at 64 days. AV11 (*trcP-glnA*) remained below the assay’s detection limit of 10^3^ CFU **·** g^−1^ soil substrate (Fig. 5A). Co-inoculation with DJ-Rif^R^ suggested that while AV6 (*glnA D49S*) might display an equivalent competitive fitness under these growth conditions, AV12 (*trcP-glnA*; *ΔnifL*) was apparently displaced by DJ-Rif^R^ at 64 days of co-inoculation (Fig. 5C).

The consumption of soil’s NH_4_^+^ by DJ-Rif^R^ was more pronounced in soil supplemented with glucose, and inoculation with AV6 (*glnA D49S*) or AV12 (*trcP-glnA*; *ΔnifL*) still resulted in a moderate accumulation of NH_4_^+^ (*p = 0.0293* and *p < 0.0001*, respectively)( Fig. 5B). NH_4_^+^ accumulation remained low in every case, with the exception of moderate peaking amounts observed in the DJ-Rif^R^ and AV6 (*glnA D49S*) and DJ-Rif^R^ and AV12 and (*trcP-glnA*; *ΔnifL*) co-cultures at 16 days, before it declined very sharply (*p = 0.0001* and *p < 0.0001*, respectively) Fig. 5D).

### “Undisturbed” soil approach

**Fig. 6:**
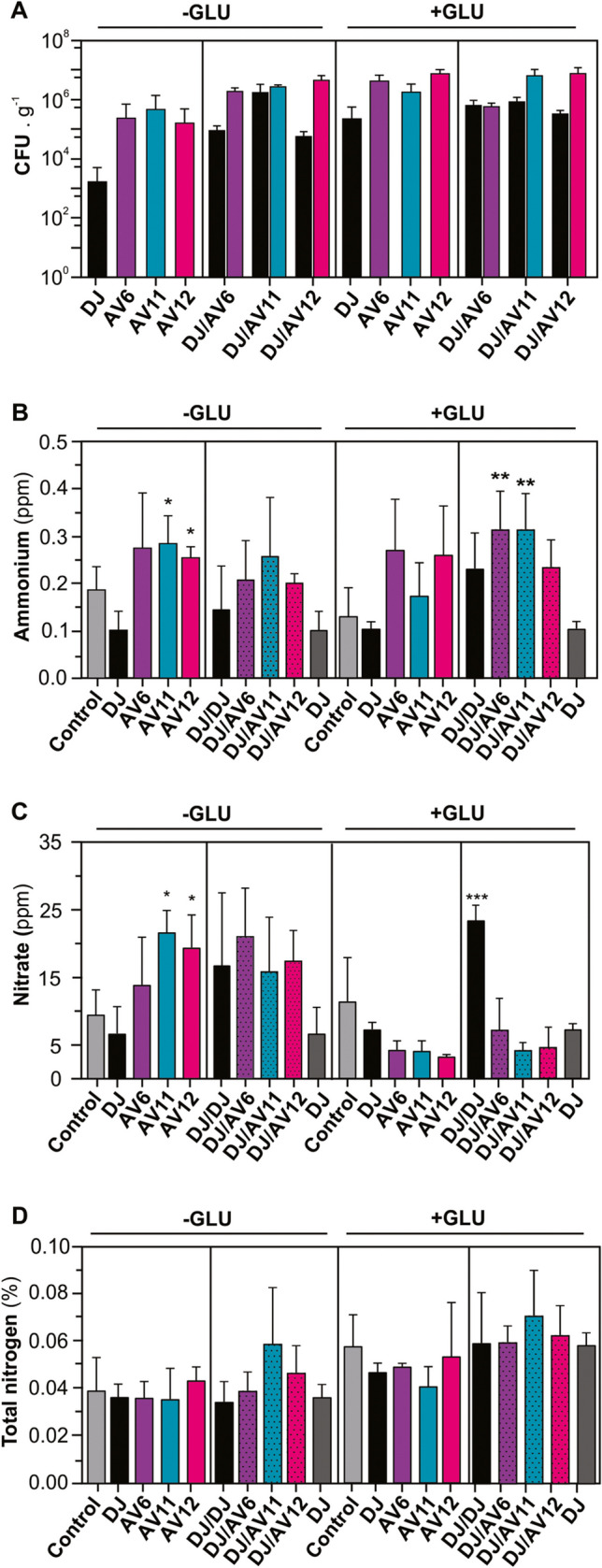
Survival, relative competitive fitness, and NH_4_^+^ release of different *A. vinelandii* mutant strains and N-speciation in “undisturbed” soil. Initial CFUs were about 2 **·** 10^7^ bacterial CFU **·** g^−1^ soil substrate. **A** Time course of CFU recovery from the soil after 28 days of inoculation. **B** Time course of NH_4_^+^ accumulation in the soil after inoculation with individual strains; hyphens (-) indicate non-inoculated soils. **C** Time course of the relative competitive fitness of *A. vinelandii* DJ-Rif^R^ and mutant strain in the soil. **D** Time course of NH_4_^+^ accumulation in soil after inoculation with combinations of *wt* and mutant strains. In **B**–**D**, samples were collected after 77 days of inoculation. Each data point represents the mean and standard deviation of three independent experiments. Results in **B**–**D** were statistically analyzed using ANOVA and Tukey as multiple comparison posttests. Asterisks (*) indicate significant differences between treatments and non-inoculated soil at each sampling time (*p ≤ 0.05; *p ≤ 0.01; and **p ≤ 0.001)

Using this experimental approach, we basically observed a similar trend in the growth performance. Glucose supplementation enabled a larger bacterial population for all the strains. All tested mutant strains appeared to display a similar fitness to the DJ-Rif^R^ strain under these experimental conditions (Fig. 6A). Consistently, with the homogenized soil approach, while inoculation with *A. vinelandii* strain DJ-Rif^R^ resulted in almost half the NH_4_^+^ content of the soil, the inoculation of mutant strains almost doubled the soil’s ammonium content, for about a threefold-higher level than the *wt* strain (*p < 0.05*) and also when co-inoculated with DJ-Rif^R^ (*p < 0.01*) (Fig. 6B). A similar profile was observed for the effect of bacterial inoculation in the NO_3_^−^ content of the soil, for up to a threefold increase after single inoculation with AV11 (*trcP-glnA*) or AV12 (*trcP-glnA*; *ΔnifL*) (*p < 0.05*). While glucose supplementation did not change the level of NO_3_^−^ accumulation by the *wt* strain, it resulted in an about fourfold-lower level for the three mutant strains tested (Fig. 6C). As a control for the competition assays, we also inoculated the “undisturbed” soil with double amount of *wt* cells to see whether the overall bacterial dose would have an effect on N content and chemical speciation. We observed a larger effect than expected in these variables, even in the accumulation of mineral-N species in the soil, which was quite pronounced for the double dose inoculation. More consistent results were observed for soil supplemented with sugar for a final accumulation of NO_3_^−^ of up to 25 ppm (Fig. 6C).

Glucose supplementation tended to increase the total N content of the soil. However, this effect appears not to be specifically related to the inoculation of *A. vinelandii.* No differences were observed among the *A. vinelandii* mutant stains (Fig. 6D).

### Genetic stability of *A. vinelandii* NH_4_^+^-releasing strains in the soil

**Fig. 7:**
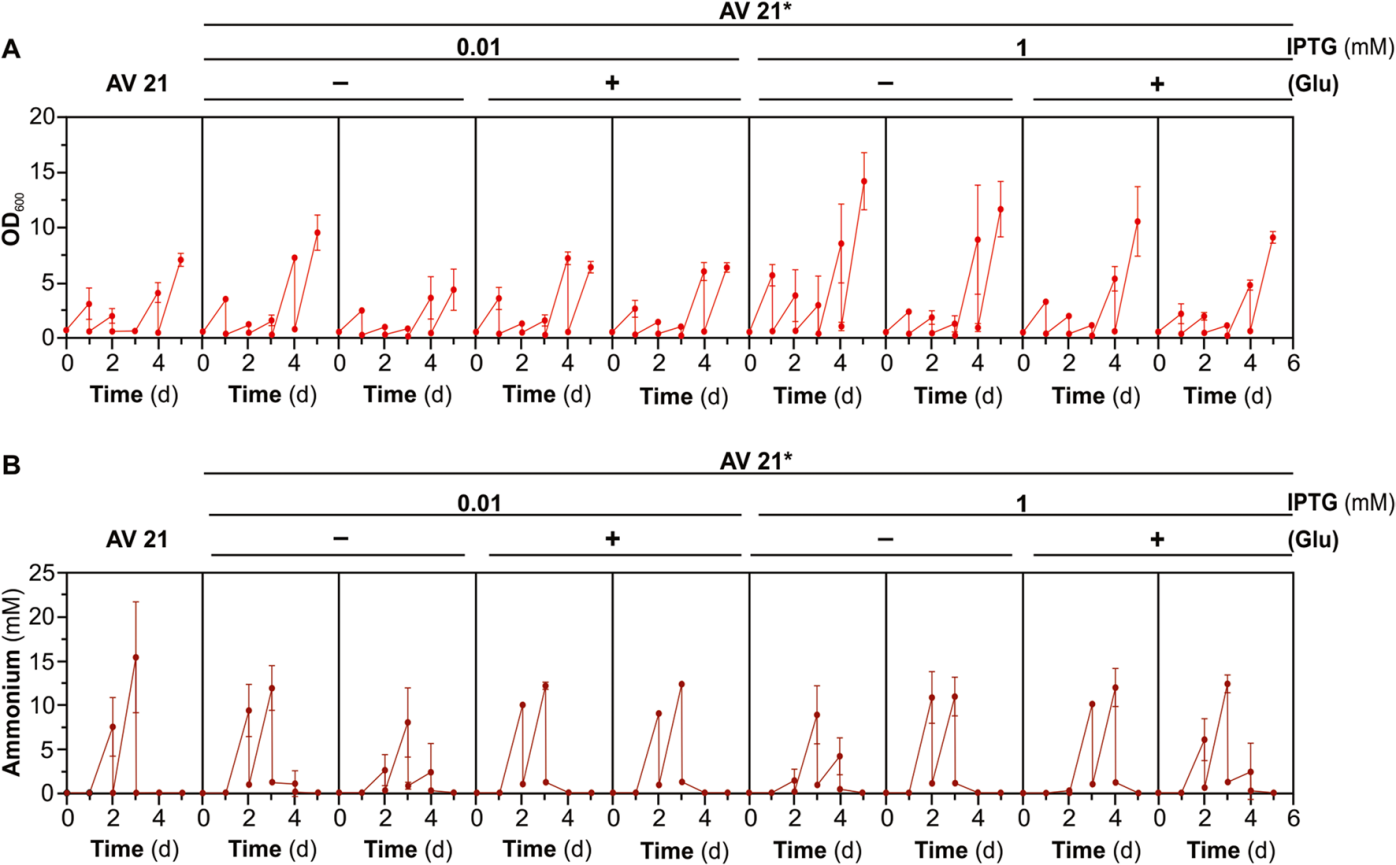
Genetic stability of NH_4_^+^-releasing phenotype of *A. vinelandii* strain AV21 (AV11-Rif^R^) in the soil. The soil was inoculated at 2 **·** 10^7^ CFU **·** g^−1^ soil with bacteria previously induced with IPTG at the indicated intensities, and bacterial clones were isolated after 64 days of incubation. A total of eight independent clones were isolated (AV21*): four clones originated from AV21 cells previously induced with 0.01-mM IPTG and four clones from cells induced with 1-mM IPTG. Within each set of four clones, two clones were provided from soil supplemented with glucose, while the other two were from non-supplemented soil. The AV21* strains were cultivated in the presence of 0.1-mM IPTG for 48 h to replenish GS levels and then subjected to an identical analysis as that shown in Fig. 2 for four consecutive sub-cultivation cycles in a regular culture medium lacking IPTG. **A** Growth (OD_600_) and **B** NH_4_^+^ accumulation at the end of each cycle. Each data point represents the mean and standard error of two independent experiments

In a previous report, we show that 12 out of the 12 strains (AV11*) isolated after sub-culturing AV11 in Burk’s medium in the absence of IPTG for 28 days displayed a fast and IPTG-independent growth and loss of the NH_4_^+^ excretion phenotypes (Ambrosio and Curatti 2021). In this study, to analyze the long-term phenotypic stability of NH_4_^+^ excretion of strain AV11 (*trcP-glnA*) in the soil, the strain was additionally labeled with a Rif^R^ marker to accurately recover the strain (AV21) because of its specific phenotype (Ap^R^, Kn^R^, Rif^R^). After 64 days of incubation using the homogenized soil approach, we isolated a total of eight independent clones for further analysis (designated as AV21*). Specifically, four clones originated from AV21 cells previously induced with 0.01-mM IPTG, while the remaining four clones were from cells induced with 1-mM IPTG. Within each set of four clones, two clones were cultivated in soil supplemented with glucose, while the other two were grown in non-supplemented soil. As Fig. 7 shows, all the clones analyzed displayed a very similar growth and NH_4_^+^-excretion phenotype to that of the parental strain AV21. The recovered phenotype was also identical to that of AV11 which reverted to faster-growing and non-ammonium-excreting cells after about 15–20 generations (Fig. 2 and Ambrosio and Curatti 2021).

Overall, these results indicated that the growth and NH_4_^+^-excreting phenotypes of these genetically modified strains tend to be more stable in the soil than in regular culture conditions for bacteria at the expense of a rich nutrient medium, and additional culture optimizations. At least in part, this could be related to the apparently lower number of cell generations that took place in the soil.

## Discussion

In addition to being a model bacterium for studying the fundamental aspects of N_2_-fixation (Martin del Campo et al. 2022), *A. vinelandii* has become a model for NH_4_^+^ release, which is a very important aspect towards the use of bacterial inoculants for sustainable agriculture (Bueno Batista and Dixon 2019). This model relied almost exclusively on highly aeriated submerged culture using Burk’s liquid medium as a reference condition, which led to the current knowledge on genetic aspects (Bueno Batista and Dixon 2019; Barney and Plunkett 2022) and culture conditions for sustaining and maximizing elevated levels of NH_4_^+^ production from nitrogenase-deregulated *A. vinelandii* strains (Plunkett et al. 2020). The underlying mechanisms for NH_4_^+^ excretion appeared to be similar in other diazotrophs such as *Pseudomonas stutzeri* (Bueno Batista et al. 2021), *Azorhizobium caulinodans* (Haskett et al. 2022), *Azospirillum brasilense* (Schnabel and Sattely 2021a), and likely other bacteria as well (Bueno Batista and Dixon 2019). Inoculation of plants under laboratory conditions with some of these genetically modified *A. vinelandii* bacteria showed some short-term beneficial effects on plant N nutrition, apparently at the expense of bacterial N_2_-fixation and NH_4_^+^ release (Ambrosio et al. 2017; Bageshwar et al. 2017; Mus et al. 2022). However, it is still doubtful to what extent this could be replicated under field conditions for a robust contribution to plant N nutrition (Bueno Batista and Dixon 2019). One of the main drawbacks is the fact that most nitrogenase-deregulated and/or GS-deficient strains tend to grow more slowly and show great genetic instability towards reversion into faster-growing clones that do not release NH_4_^+^ and tend to overgrow the parental mutant strains (Ambosio and Curatti 2021) under laboratory conditions. This instability was also observed in *A. brasilense* (Schnabel and Sattely 2021a; Schnabel and Sattely 2021b). Unregulated expression of the *nif* genes in recombinant *P. protegens* Pf-5 resulted in NH_4_^+^ release and N-fertilization of plants. However, it also resulted in a fitness burden on the cells, which led to a rapid decline of the bacterial population in the soil (Setten et al. 2013; Fox et al. 2016).

Moreover, from a technological point of view, poor growth of NH_4_^+^-excreting bacteria would not only compromise its effectiveness at the farm level but also at the inoculant production stage (Ryu et al. 2020).

Thus, it appears that free-living and endophytic diazotrophic bacteria make use of sophisticated signal transduction mechanisms for N homeostasis on increasing their competitive fitness in the environment, which conflicts with the increased release of NH_4_^+^ (Bueno Batista et al. 2021). Alternative genetic approaches were used to ameliorate and/or partially address this hurdle. For example, increasing the gene dose of a unidirectional adenylyltransferase variant which increased N_2_-fixation and NH_4_^+^ release via GS deactivation proved to be less prone to revert to non-NH_4_^+^-releasing clones. However, inoculation of *Zea mays* in N-poor, non-sterile soil did not improve plants’ N nutrition over the performance of the *wt* strain, suggesting that excessive fitness defects caused by extreme GS inhibition diminished strain health, resource competition, and/or colonization capacity in soil (Schnabel and Sattely 2021b). Alternatively, the introduction of the E356K mutation in *A. vinelandii* NifA resulted in unregulated overexpression of nitrogenase, NH_4_^+^ release, and no growth penalty on complex media or in minimal media supplemented with some carbon sources, under otherwise regular culture conditions (Bueno Batista et al. 2021).

This study contributed a more in-depth study in comparing different genetic strategies for NH_4_^+^ release and the comparison of their fitness burden for the desired task in regular culture medium, as most previous studies did (Bueno Batista and Dixon 2019), and also in mildly autoclaved soil. Both for much longer times than most of the previous studies did. Overall, AV12 (*trcP-glnA*; *ΔnifL*) appeared to be the most stable strain of this study in terms of growth and NH_4_^+^ release over time.

We analyzed the competitive fitness of *A. vinelandii* strains with different mutations and combinations of mutations on *nifL* and *glnA.* A third strategy based on a mutation on the NH_4_^+^/ CH_3_NH_3_⁺ transporter encoded by the *amtB* gene was also reported (Barney et al. 2015). However, in contrast to modifications on *nifL* or *glnA*, which resulted in an NH_4_^+^ accumulation in the spent medium at millimolar levels, *amtB* mutant strains only accumulated in the low micromolar range (Barney et al. 2015). Nevertheless, *amtB* mutant strains cross-feed fixed N to sustain the growth of a non-N_2_-fixing microalga in the absence of an N source other than air (Barney et al. 2015), at least to a similar extent to *nifL* (Ortiz-Marquez et al. 2012) or *glnA* (Ortiz-Marquez et al. 2014; Ambrosio et al. 2017) mutant strains. This suggests that the maximum concentration of NH_4_^+^ a mutant strain can accumulate in the culture medium would not necessarily be an accurate probe for successful cross-feeding or biofertilization purposes. Additionally, deletion mutations on *amtB* might be less prone to genetic reversion than regulatory mutations on genetic elements for which loss-of-function mutations tend to arise at high frequency leading to phenotypic reversion. Thus, it would be interesting to also consider *amtB* mutant strains in future studies on the phenotypic stability of NH_4_^+^ release.

We co-cultivated each mutant strain together with the *wt* strain in an attempt to force competition for presumably the same resources and to enable a more accurate quantification of the fitness penalty for high NH_4_^+^ release. First, co-culturing was conducted in a rich liquid medium (Burk’s medium, as a reference culture condition) for up to seven successive sub-cultivation runs with replenishment of nutrients and removal of the accumulated NH_4_^+^. This approach allowed us to analyze the mutant strains’ competitive fitness under a broad dynamic range of growth penalties for NH_4_^+^ release. It is likely that this experimental approach would represent a low competition environment to disclose even low differences in burden for high NH_4_^+^ release which can only be observed following the bacterial population dynamics longer than a single-growth curve analysis. It became clear that, sooner or later, all the strains lost the ability the release NH_4_^+^ by the 7th sub-cultivation cycle. Some strains, most noticeably the double mutant strain AV7, which is deregulated for both N_2_-fixation (*nifL*) and N-assimilation (*glnA*), presented a severe growth penalty and a remarkable tendency to revert to faster-growing clones at longer times. On the other hand, it is important to highlight that strain AV3, which is only deregulated for N_2_-fixation (*nifL*), grows almost as fast as the *wt* strain for up to three consecutive sub-cultivation runs and showed an equivalent competitive fitness for up to two consecutive sub-cultivation cycles but remains below the assays detection limit by the 7th sub-cultivation cycle.

Unpredictably, strains with deregulated GS imposed strong competition to the *wt* strain, for a decrease of its population of up to 10,000-fold by the second day (Fig. 3 and Fig. S1). The higher the GS accumulation in the cells before the competition assay, the higher its relative competitive fitness. As expected, because the competition assays were conducted in the absence of the *glnA* gene inducer, GS depletion by cell division resulted in a reversion of the relative competitive fitness towards the *wt* strain. The mechanism underlying this effect is not understood at this moment.

As expected, the co-cultivation of NH_4_^+^-excreting strains together with the *wt* strain resulted in a pronounced reduction in the accumulation of NH_4_^+^ in the culture medium. Although not confirmed in the study, it is possible that the *wt* strain, as most microbes would do in the soil, would represent a sink for the NH_4_^+^ released by the engineered strains. Because it appears that NH_4_^+^ loss from the *A. vinelandii* cells passively follows a gradient concentration from cellular (higher) to extracellular (lower) (Brewin et al. 1999), it is possible that increasing the gradient would favor more N_2_ capture from the air and to hold it in the soil as a reservoir of organic N, which can eventually be accessible to plants nutrition. Fixed N chemical speciation in the soil is a key component for success towards efficient plant bio-fertilization. For example, inoculation of cucumber plants in sterile substrate with high doses of the high NH_4_^+^-excreting strain AV12 resulted in an apparent plant toxicity (Ambrosio et al. 2017).

To analyze whether knowledge from previous experiments conducted under typical culture conditions for *A. vinelandii* would still apply in conditions resembling a soil environment, we challenged the strains to compete for thriving in mildly autoclaved soil for more than 2 months. This time frame was chosen as a reference because it represents the time around the maximum demand for N of corn plants around developmental stage V6 (6–8 weeks) (Abendroth et al. 2011). Although not directly addressed in this study, the ultimate expectation is that matching the timing and dose of N supplied from N_2_-fixing inoculants to crop uptake would favor N use efficiency, as it is required for both economic and environmental sustainability (Salim and Raza 2020).

As expected, but not experimentally demonstrated until now, contrary to the regular culture medium assays, *A. vinelandii* did not grow much in mildly autoclaved soil and the environmental-like conditions used in this study. However, with the expected exception of the conditional mutant strains AV11 and AV12 at longer times, the bacterial population remained quite stable for up to 2 months of incubation. While this observation significantly advances the state-of-the-art in our research, it is crucial to consider the impact of simplifying the experimental system’s complexity, even by mild soil autoclaving. Under our experimental conditions, soil treatment using autoclaving at 121 °C for 20 min leads to abundant growth of other bacteria and fungi, especially after 1 month of treatment. This occurs regardless of using a somewhat selective culture medium for plate counting of *A. vinelandii*’s CFUs. This situation conditioned the maximum incubation time in the soil to accurately identify *A. vinelandii* in each experiment. As a reference, Li et al. (2023) demonstrated that autoclaving soil at 121 °C in wet cycle mode for 45 min resulted in the sterilization of 86.75% of soil bacteria and a similar amount of fungi. Interestingly, another study reported greater survival of *Escherichia coli* after inoculation in autoclaved soils (which were partially sterilized) compared to natural soils. This suggests that certain soil microorganisms, reduced by heat treatments, likely contributed to the persistence of *E. coli* in the soil. Furthermore, the same study revealed that soil tends to recover its original biodiversity in a temperature-dependent manner following perturbation (Baker et al. 2020). Additionally, heating could alter soil properties such as pH, organic matter content, and particle size distribution (Li et al., 2023). These changes may directly or indirectly influence the survival of a bacterium inoculated in partially sterilized soil.

Despite using partially sterilized soil, the persistence of *A. vinelandii* was unexpectedly high. This is particularly remarkable given the genetic background of the parental strain. The laboratory model strain DJ, a highly genetically transformable variant (Setubal et al. 2009), was derived from strain OP—an isolated non-gummy variant of *A. vinelandii*. Strain DJ has played a crucial role in physiological, biochemical, and genetic research on N_2_ fixation over the past eight decades (Bush and Wilson 1959), including the set of mutant strains used in our current research. A recent study on *Azotobacter spp.* strains freshly isolated from wheat rhizosphere showed a great tendency of most strains to produce copious amounts of exopolysaccharides (EPS) and to form biofilms upon salt stress and P scarcity. Thus, it was proposed that living in EPS-rich biofilms might be a preferred lifestyle for the genus in the soil for helping to cope with stress factors such as salinity, drought, and suboptimal temperatures (Çam and Bicek 2023). Thus, it is likely that the outreach of our study can still be limited because of the specific characteristics of the genetic background used, which might underscore the actual fitness of *A. vinelandii* in the soil.

At least in part as a consequence of slow growth, the burden of high NH_4_^+^-release in terms of competitive fitness in comparison to the *wt* strain was mostly negligible for up to 1 month. As expected, in the absence of the inducer IPTG, the survival of AV11 and AV12 strains was compromised. Moreover, although we expected some genetic reversion into faster growing and non-NH_4_^+^-releasing clones at longer times, as demonstrated in the regular culture conditions, apparently this was not the case. To confirm that, we re-isolated several clones after 2 months of inoculation into the soil and observed that all of them conserved the original growth and NH_4_^+^-releasing phenotypes. This contrasts largely with our previous observation of genetic/phenotypic instability of AV11 and AV12 strains under regular culture conditions after about 15–20 bacterial cell generations (Ambrosio and Curatti 2021).

Nevertheless, even at these slow (or even non-dividing states) all strains proved to be metabolically active. While apparently, the *wt* strain tends to convert NH_4_^+^ soil into organic-N (likely as microbial biomass), most genetically engineered strains not only increase total N in the soil but still increase NH_4_^+^ and NO_3_^−^ at 2 months of inoculation. While the NO_3_^−^ in the non-inoculated pots was about 9 ppm, which is regarded as a non-fertile condition for corn farming, inoculation with strain AV11 buildup the soil’s NO_3_^−^ to 22 ppm. This is certainly very positive since a large meta-analysis indicated that NO_3_^−^ concentrations could explain 82% of the variability in relative yields of corn. More specifically, the study indicated that 21 ppm NO_3_^−^-N in the surface l-ft layer of soil is adequate to attain maximum yields and suggested that a range of 20 to 25 ppm should be considered optimal (Blackmer et al. 1989). The assay we conducted did not distinguish between NO₃⁻ in the soil and that accumulated in the microbial biomass. Consequently, we cannot definitively determine whether the inoculated bacteria directly influence changes in soil NO₃⁻ content. However, we can infer that soil inoculation with strains possessing varying abilities to release NH₄⁺ resulted in differential alterations in the soil’s NO₃⁻ pool. Since *A. vinelandii* is not a nitrifying bacterium, the conversion of NH₄⁺ into NO₃⁻ must be attributed to nitrifying microorganisms (Hayatsu et al. 2021) that survived the soil treatment by mild autoclavation. The primary environmental factors influencing nitrification in agricultural soils include agricultural management practices, pH, ammonia concentration, temperature, and water content (Hayatsu et al. 2021). The observation of NO_3_^−^ accumulation in “undisturbed” soil supplemented with glucose of up to 25 ppm after inoculating with double the amount of *wt* cells (*p < 0.001*) was not expected, and it is currently not completely understood. This increases the level of complexity for the understanding of the physiology of these bacterial populations in the soil. As a working hypothesis for future studies, we are tempting to propose that soil NH_4_^+^ depletion by a larger population of *wt A. vinelandii* can switch cells into diazotrophic growth sooner, which under the scarcity conditions of some key nutrients in the unfertile soil would trigger unbalanced growth, NH_4_^+^ release, and also nitrification. This effect could increase the N content of the soil transiently. Thus, future research would need to consider a closer inspection of bacterial dose and time for a clearer appreciation of changes in the N content and chemical speciation in the soil after inoculation.

Sugar addition into the soil in simulating rizo-depositions somehow shaped the bacterial population dynamics. We observed that the *wt* strain appeared to benefit from this addition more than the recombinant strains. Diminished sugar uptake by *A. vinelandii* strains with lower GS activity under regular culture conditions was demonstrated before (Ortiz-Marquez et al. 2014). Thus, surpassing an apparent carbon limitation, most strains, and especially the *wt* strain become a stronger sink for soil’s mineral-N, including the NH_4_^+^-released by the recombinant strains, and largely changed the speciation of N in the soil towards a larger pool of organic-N. Other nutritional limitations, and/or environmental characteristics, could have hidden an even better performance of these strains in the soil. For example, it was shown that P and Mo co-limit asymbiotic BNF in tropical forests (Wurzburger et al. 2012). Also, an agriculture-targeted comprehensive meta-analysis showed a very high yield response to N_2_-fixing inoculants (up to almost ≥ 90.0%), only under a minimum P content of 45–75 kg **·** ha^−1^ (Schütz et al. 2018).

Even though the heterogeneity of the bacterial distribution in the “undisturbed” soil experiments, the results showed herein were very robust for the assessment of the bacterial populations. However, results for N content and speciation tended to be more variable, likely reflecting different metabolic activities at the microscale, regardless of the presence of the bacteria. These would warrant further research to capture more accurately the details of this feature in specially designed experiments.

This study represents a step forward that contributes to bridging a gap in knowledge between laboratory studies and the expected performance of bio-inoculants in the soil. We presented an analysis of the effect of the burden of the genetic improvement of NH_4_^+^ release on *A. vinelandii*’s competitive fitness and efficiency at increasing the N available to crops in the soil. Intensification of research, including microbial communities relevant at the farm level and plants and regulatory constraints for using genetically modified bacteria will be needed before a more direct extrapolation of knowledge to commercial inoculants can be more conclusively made.

## Supplementary Information

Below is the link to the electronic supplementary material.

Supplementary file1 (PDF 628 KB)

## Data Availability

Data not included within the manuscript is available upon written request from the corresponding author.
