# Supplementary material for: Competitive fitness and stability of ammonium-excreting Azotobacter vinelandii strains in the soil
Source: Appl Microbiol Biotechnol. 2024 Jun 18;108(1):378. doi: 10.1007/s00253-024-13231-1 (PMC11189346; doi:10.1007/s00253-024-13231-1)
Supplement: Supplementary file 1 — Supplementary file1 (PDF 628 KB) [file 253_2024_13231_MOESM1_ESM.pdf]

Supplementary material

Competitive fitness and stability of ammonium-excreting *Azotobacter vinelandii* strains in the soil

Rafael Ambrosio[1], Gonzalo Burgos Herrera[1], Mauro Do Nascimento[1], Luciana Anabella Pagnussat[1,2] and Leonardo Curatti[1]*

[1] Instituto de Investigaciones en Biodiversidad y Biotecnología – Consejo Nacional de Investigaciones Científicas y Técnicas, Mar del Plata, Buenos Aires, Argentina and Fundación para Investigaciones Biológicas Aplicadas

[2] Facultad de Ciencias Agrarias, Universidad Nacional de Mar del Plata, Balcarce, Buenos Aires, Argentina

*Corresponding author

Leonardo Curatti. Address: Instituto de Investigaciones en Biodiversidad y Biotecnología (INBIOTEC), Vieytes 3103, Mar del Plata (7600), Argentina. Tel.: +54 223 410 2560; Fax: +54 223 475 7120. E-mail address: lcuratti@inbiotec.conicet.gov.ar (L. Curatti).

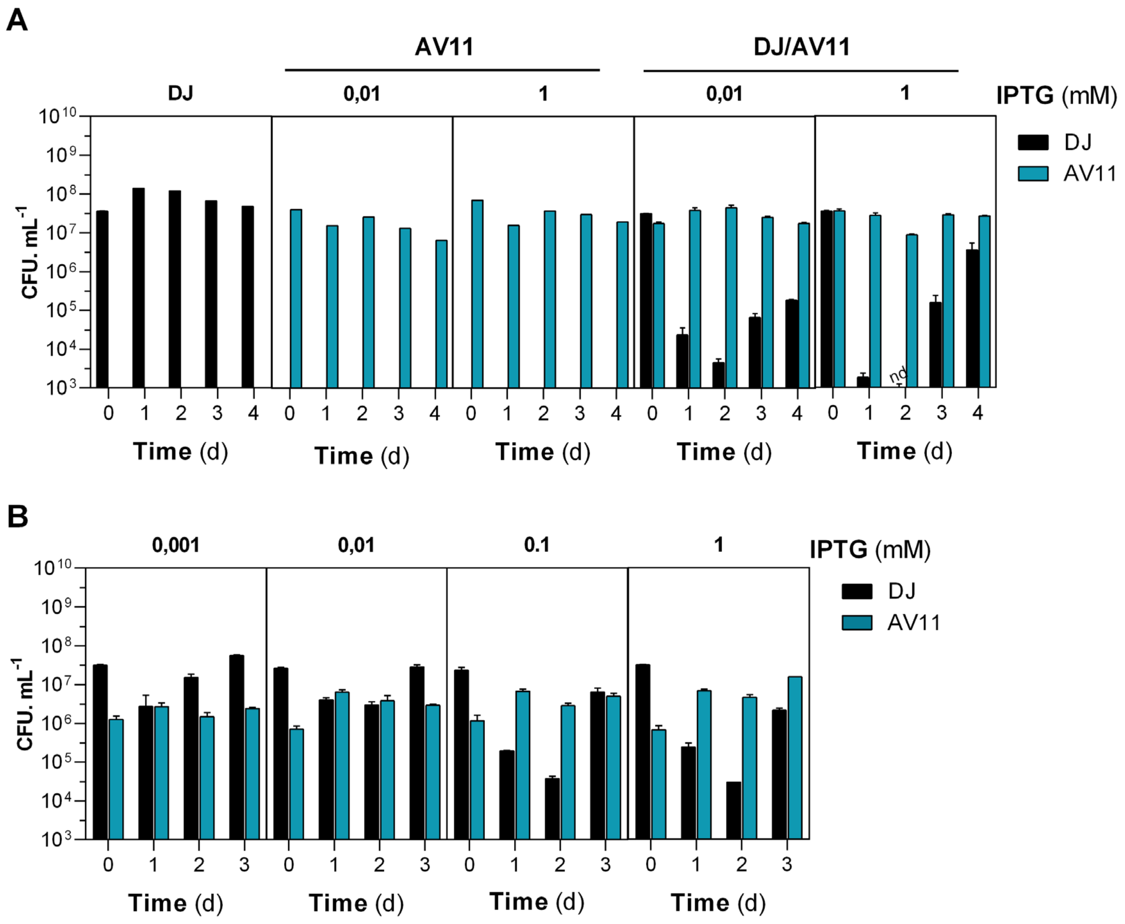

Supplementary Fig. S1. Relative competitive fitness of *A. vinelandii* AV11 (*trcP-glnA*) mutant strain and the DJ-Rif^R (*wt*) strain in regular culture medium. A) CFU determinations at the beginning and at the end of cycles 1 to 4. These experiments were equivalent to that shown in Fig. 3C, but the initial CFU (about $5 \cdot 10^7 \cdot mL^{-1}$) were more even. Each data point represents the mean and standard error of two independent experiments. B) CFU determinations at the beginning and at the end of cycles 1 to 3. This experiment was also equivalent to those shown in Figs. 3C and Suppl. Fig. S1, but a broader range of *glnA* pre-induction strength was used, and the initial CFU were about $5 \cdot 10^7 \cdot mL^{-1}$ and $1 \cdot 10^6 \cdot mL^{-1}$ for DJ (*wt*) and AV11 (*trcP-glnA*), respectively. Each data point represents the mean and standard deviation of three independent experiments. nd indicates $\leq 1 \cdot 10^3$ CFU $\cdot mL^{-1}$.
